# Chronic Cystic Mastitis

**Published:** 1914-05

**Authors:** 


Chronic Cystic Mastitis. E. S. Judd, of Rochester, Minn., *Jour. Mich. State Med. So.*, January, 1914, reports that up to 1913, 929 cases of breast tumor were operated on at the Mayo Clinic, 711 being definitely cancerous but most of them showing evidence of chronic cystic mastitis in varying degree. 218 were definitely classified as chronic cystic mastitis. 11 of these occurred in males, 5 in each breast and 1 in both breasts. Of the 207 females, 140 were parous, 45 nulliparous, 3 had miscarriages (sic) 22 not mentioned. Seventy-nine per cent. of the strictly cancerous tumors occurred in the "cancer age," 30-60, while 85.3% of the non-malignant cases occurred in this period,

